# Liquid–Liquid Phase Separation in Major Hallmarks of Cancer

**DOI:** 10.1111/cpr.70122

**Published:** 2025-09-19

**Authors:** Chen‐chen Xie, Ting Wang, Xin‐ran Liu, Yan Wang, Qin Dang, Tian Ding, Jia‐qi Xu, Xian‐jun Yu, Hai Lin, Xiao‐wu Xu, Yi Qin

**Affiliations:** ^1^ Department of Pancreatic Surgery Fudan University Shanghai Cancer Center Shanghai China; ^2^ Department of Oncology, Shanghai Medical College Fudan University Shanghai China; ^3^ Shanghai Pancreatic Cancer Institute Shanghai China; ^4^ Shanghai Key Laboratory of Precision Medicine for Pancreatic Cancer Shanghai China; ^5^ Pancreatic Cancer Institute, Fudan University Shanghai China; ^6^ Department of Pancreatic Surgery, Digestive and Vascular Surgery Center The First Affiliated Hospital of Xinjiang Medical University Urumqi Xinjiang China

**Keywords:** biomolecular condensates, cancer, cancer therapeutics, dysregulated state, liquid–liquid phase separation

## Abstract

The malignant transformation of cancer cells is underpinned by the dysregulation of essential cellular processes, including genome stability maintenance, DNA repair, transcriptional control and signal transduction. These processes are not randomly distributed but are spatiotemporally coordinated through dynamic molecular assemblies. Recent advances have highlighted the pivotal role of biomolecular condensates, membraneless compartments formed via liquid–liquid phase separation (LLPS), in compartmentalising and regulating these key functions. LLPS enables the concentration and organisation of proteins and nucleic acids, creating distinct biochemical environments that facilitate cellular decision‐making. Importantly, aberrant phase separation has been increasingly implicated in the acquisition of cancer hallmarks, such as sustained proliferative signalling, resistance to cell death and immune evasion. In this review, we summarise the physicochemical principles of LLPS, examine its emerging roles in oncogenic transformation and discuss the therapeutic potential of targeting phase separation in cancer. Our findings highlight LLPS as a novel and versatile regulatory layer in tumour biology and an emerging frontier in precision oncology.

## Graphical abstract

Aberrant condensates formed through phase separation are involved in the dysregulation of various critical cellular processes, including genome stability, transcriptional regulation and signal transduction, thereby promoting malignant transformation and the acquisition of multiple cancer hallmarks.

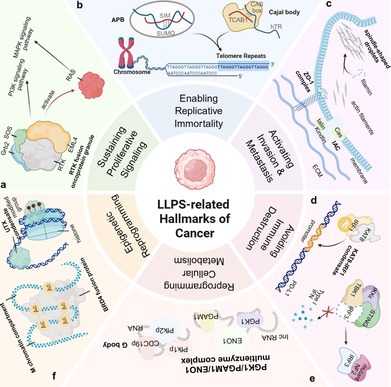

**Abbreviations**
ALS: amyotrophic lateral sclerosis
ALT: alternative lengthening of telomeres
APBs: ALT‐associated promyelocytic leukaemia bodies
AR: androgen receptor
ATPS: aqueous two‐phase system
CAR: Chimeric antigen receptor
CBs: Cajal bodies
CTCs: circulating tumour cells
CTD: C‐terminal domain
DBD: DNA‐binding domain
DFCs: dense fibrillar components
DOX: doxorubicin
ECM: extracellular matrix
EMT: epithelial‐to‐mesenchymal transition
ERα: oestrogen receptor alpha
FCs: fibrillar centres
FRAP: fluorescence recovery after photobleaching
G4: G‐quadruplex
GCs: granular components
H3K4me1: monomethylation of histone H3 at lysine 4
HDAC6: histone deacetylase 6
IACs: integrin adhesion complexes
IDRs: intrinsically disordered regions
LBD: ligand‐binding domain
LCDs: low‐complexity domains
LLPS: liquid–liquid phase separation
MLO: membranelles organelle
NTD: N‐terminal domain
PLD: prion‐like domain
PTMs: posttranslational modifications
PXN: paxillin
RBD: RNA‐binding domain
ROS: reactive oxygen species
RRM: RNA recognition motif
RTKs: receptor tyrosine kinases
SGs: stress granules
TCR: T‐cell receptor
TERRA: telomeric repeat‐containing RNA
TJ: tight junction
TME: tumour microenvironment
TMMs: telomere maintenance mechanisms

## 1 Introduction

Cancer is a complex and multifaceted disease characterised by a series of malignant capabilities that promote the transition from normal cells to tumour cells. The ‘Hallmarks of Cancer’ framework, introduced by Hanahan and Weinberg in 2000, originally outlined six fundamental traits acquired during tumorigenesis, including sustaining proliferative signalling, enabling replicative immortality, activating invasion and metastasis and other traits [1]. This model was further expanded in 2011 and updated again in 2022 to encompass novel concepts such as reprogramming energy metabolism, avoiding immune destruction and non‐mutational epigenetic reprogramming, thereby reflecting the complexity and adaptability of cancer biology in modern understanding [2, 3]. Fundamentally, these characteristics arise from genetic mutations and the dysregulation of cellular processes [4, 5, 6]. Accumulating evidence highlights the pivotal role of dysregulated liquid–liquid phase separation (LLPS) in cancer biology.

Phase separation refers to the process by which biomolecules, such as proteins and nucleic acids, separate from a homogeneous solution through interactions, forming dense and sparse phases. In cells, these phases typically exist in a liquid state, commonly referred to as LLPS [7, 8, 9].

LLPS is driven by weak multivalent interactions between molecules or within molecules, leading to the formation of membraneless compartments that are segregated from the surrounding environment. These micron‐scale liquid compartments, termed biomolecular condensates or membraneless organelles (MLOs), compartmentalise cellular functions without physical membranes [10, 11, 12]. Biomolecular condensates are higher‐order molecular assemblies formed by biological macromolecules, enabling cells to achieve biochemical compartmentalisation without requiring membranes [11]. These condensates include P‐bodies [13, 14], Cajal bodies (CBs) [15], nucleoli [16], stress granules (SGs) [17], PML bodies [18] and superenhancers [19], all of which participate in diverse cellular processes. By concentrating functionally related macromolecules such as proteins and nucleic acids, these condensates provide dedicated regions for biochemical reactions and enable fine temporal and spatial regulation through their rapid assembly and disassembly.

**FIGURE 1:**
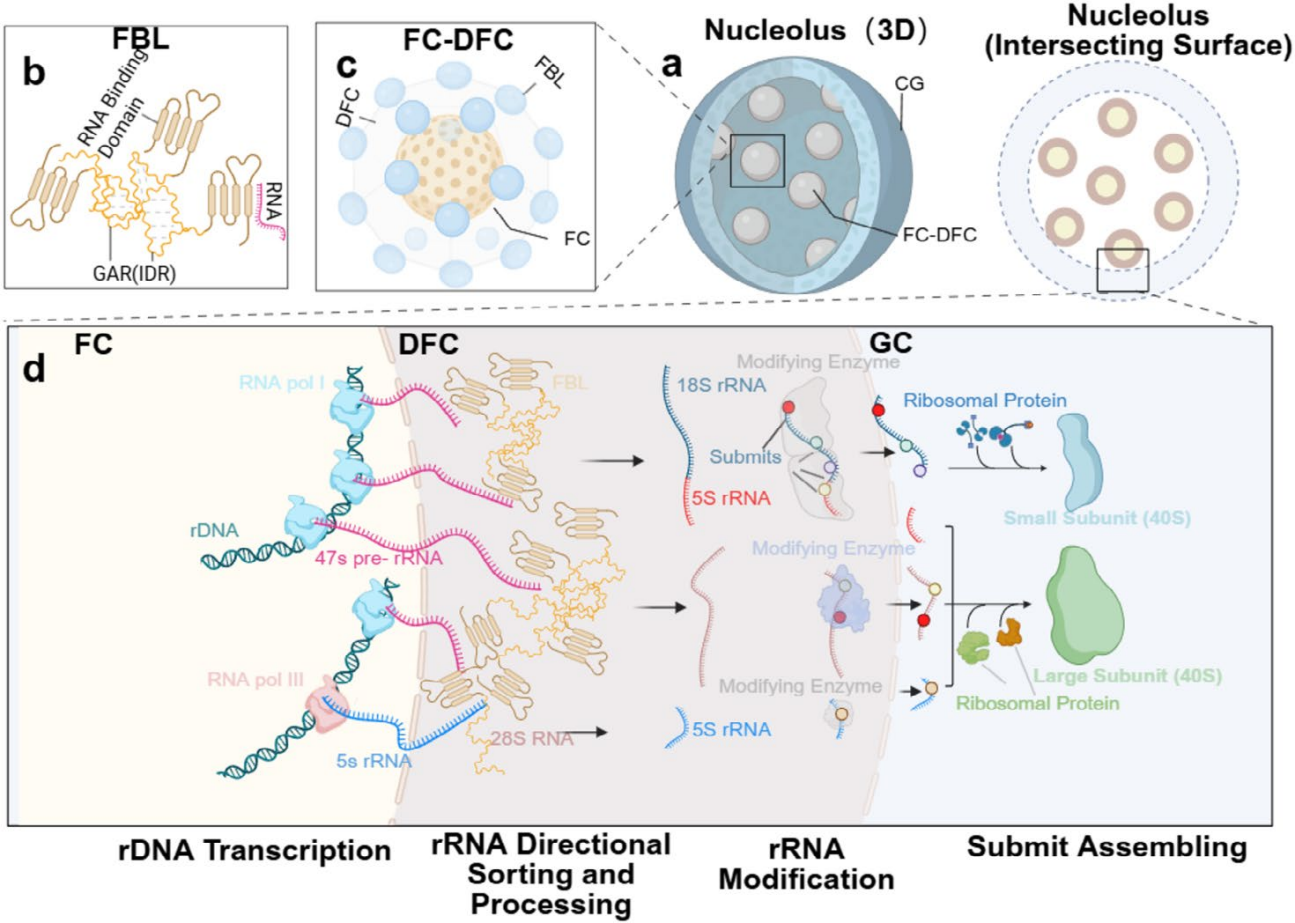
The nucleolus as a multiphase biomolecular condensate and its role in rRNA processing. (a) The nucleolus is composed of three distinct subcompartments with different compositions and functions: the fibrillar centre (FC), the dense fibrillar component (DFC), and the granular component (GC). The GC, enriched with nucleolar proteins, forms a liquid‐like ‘shell’ that encloses the central ‘core’ composed of the DFC and FC, thereby creating a multiphase condensate. (b) Fibrillarin (FBL) undergoes self‐association through its GAR domain and binds to the 5′ end of nascent rRNA transcripts via its RNA‐binding domain. (c) A spherical network is formed by the assembly of 18–24 regularly spaced FBL condensates, constituting the DFC, which surrounds the central FC. (d) The transcription and processing of nascent rRNA primarily occur within distinct functional regions of the nucleolus. Within the FC, rRNA transcription is mediated by two different RNA polymerases: Pre‐47S RNA is transcribed by RNA polymerase I, whereas 5S RNA is synthesised by RNA polymerase III. These nascent rRNA precursors are subsequently transported to the DFC under the guidance of FBL clusters for further processing. In the DFC, pre‐47S RNA undergoes a series of precise processing steps, ultimately being cleaved into 18S RNA, 5.8S RNA and 28S RNA. These rRNAs, along with 5S RNA, are chemically modified by specific modifying enzymes. Following modification, the rRNAs are transported to the GC for ribosomal subunit assembly. Within the GC, 18S RNA associates with corresponding ribonucleoproteins to form the small ribosomal subunit, whereas 5.8S RNA, 28S RNA and 5S RNA, in coordination with their respective ribonucleoproteins, assemble to form the large ribosomal subunit. Finally, these mature ribosomal subunits are transported to the nucleoplasm, where they participate in subsequent protein synthesis processes.

However, the simplest physical picture of a homogeneous liquid phase is often not enough to capture the full complexity of intracellular condensates. Recent studies have revealed that some condensates exhibit a multistate structure, with subregions that differ in maturation characteristics and function [20]. The nucleolus of mammals represents one of the most extensively studied multiphase condensates, comprising fibrillar centres (FCs), dense fibrillar components (DFCs) and granular components (GCs), each with distinct biochemical roles in ribosome biogenesis [21, 22] (Figure 1).

Studies using high‐resolution imaging and other methods to determine molecular composition have demonstrated that, although membraneless compartments can vary in their location, composition and function, they generally follow similar dynamics and assembly pathways [23]. A defining characteristic of biomolecular condensates is their liquid‐like nature [24, 25]. Taking P‐bodies as an example, these biomolecular condensates exhibit droplet formation under applied shear stress, undergo fusion upon contact and display distinct ‘wetting’ behaviour when interacting with surfaces such as the nuclear envelope [20]. Another manifestation of this liquid‐like property is the high mobility of molecules, which facilitates rapid exchange of internal components with the surrounding medium on timescales ranging from seconds to minutes, as evidenced by fluorescence recovery after photobleaching (FRAP) experiments [25, 26]. These characteristics enable the biomolecules to achieve localised positioning and maintain dynamic exchange with their environment, thereby allowing condensates to [11]:

- Resist interference: Form phase boundaries that isolate biochemical reactions from external disruptions, thus buffering against cellular noise.
- Regulate metabolic flux: Enhance enzyme and substrate activity by concentrating them or suppress activity via sequestration.
- Sense stimuli and switch states: Enable rapid sensing and response to diverse intracellular/extracellular stresses and stimuli, ensuring faster reactions than transcriptional or translational activation.

Physiological LLPS maintains cellular homeostasis. However, its dysregulation can generate aberrant condensates that disrupt signalling, gene expression and stress responses—all of which are relevant to cancer pathogenesis. For instance, cancer‐associated SHP2 mutations endow the protein with aberrant LLPS capacity, facilitating the formation of condensates that hyperactivate the MAPK signalling pathway by recruiting and activating wild‐type SHP2, thereby contributing to oncogenic signal amplification [27]. Furthermore, the loss of physiologically essential condensates can contribute to tumorigenesis as well. In fibrolamellar carcinoma, disruption of RIα biomolecular condensates by the DnaJB1‐PKA_cat_ fusion oncoprotein abolishes cAMP compartmentalisation, leading to aberrant PKA signalling hyperactivation and tumorigenesis [28]. From a diagnostic perspective, these aberrant biomolecular condensates hold potential as cancer biomarkers for detection and prognosis.

The dynamic assembly of biomolecular condensates and their exchange with the surrounding dilute phase is highly sensitive to physicochemical conditions such as pH, temperature and molecular crowding [29, 30]. Theoretically, any factor that influences the physicochemical properties of condensates has the potential to alter their functionality, thereby offering opportunities for therapeutic interventions targeting diseases caused by aberrant phase separation. Disrupting abnormal condensates through modulation of key molecular components or environmental factors may offer new strategies for selectively targeting cancer cells. Additionally, the selective condensation properties of biomolecular condensates based on LLPS have inspired the design of anticancer drug delivery systems. These systems employ the inherent phase‐separation‐driven compartmentalisation to encapsulate therapeutic agents and harness phase separation‐related stimuli‐responsive mechanisms for drug release, thereby enhancing drug bioavailability and therapeutic efficacy.

In this review, we outline the fundamental mechanisms of LLPS and examine its emerging roles in orchestrating hallmark cancer processes. Furthermore, given the pivotal role of biomolecular condensates in tumour development, this review summarises recent advances in their diagnostic and therapeutic applications. Specifically, we discuss strategies targeting aberrant condensates by disrupting or modulating their phase separation properties, exemplified by the use of EPI‐series small‐molecule inhibitors in castration‐resistant prostate cancer, which covalently bind to the androgen receptor (AR) and suppress its transcriptional activity [31]. In diagnostics, bioinformatic analyses have identified LLPS‐associated, cancer‐specific genes, such as HOXA13, TEAD4 and three others, as prognostic and immunological biomarkers in clear cell renal cell carcinoma, with their expression profiles emerging as promising tools for patient stratification [32]. Moreover, LLPS‐inspired carriers, including synthetic polypeptoid‐based delivery systems and aqueous two‐phase system (ATPS)‐derived platforms, have been engineered as tumour‐specific drug delivery systems to enhance therapeutic efficacy [33].

## 2 Fundamental Principles of LLPS

### 2.1 Thermodynamic Principles of LLPS

LLPS arises from thermodynamic principles, whereby a closed system tends to evolve towards a steady‐state configuration with minimal free energy [34].

In an ideal solution where no intermolecular interactions are present, the components mix purely under entropic driving forces. As the mixing ratio changes, the system's free energy of mixing (Δ*G*_mix_) decreases monotonically with increasing degree of mixing, reaching its minimum at the fully mixed state. Correspondingly, the chemical potential varies monotonically with concentration, maintaining a thermodynamically stable, homogeneous distribution of components.

However, in real biological systems, particularly when attractive interactions exist between molecules, the free energy of mixing (Δ*G*_mix_) must account for both entropic contributions (Δ*S*_mix_, reflecting the degrees of freedom for molecular spatial distribution) and enthalpic contributions (Δ*H*_mix_, reflecting intermolecular interactions such as hydrophobic forces and electrostatic interactions), as described by the equation Δ*G*_mix_ = Δ*H*_mix_ − *T*Δ*S*_mix_, where *T* is the absolute temperature of the system [35, 36].

**FIGURE 2:**
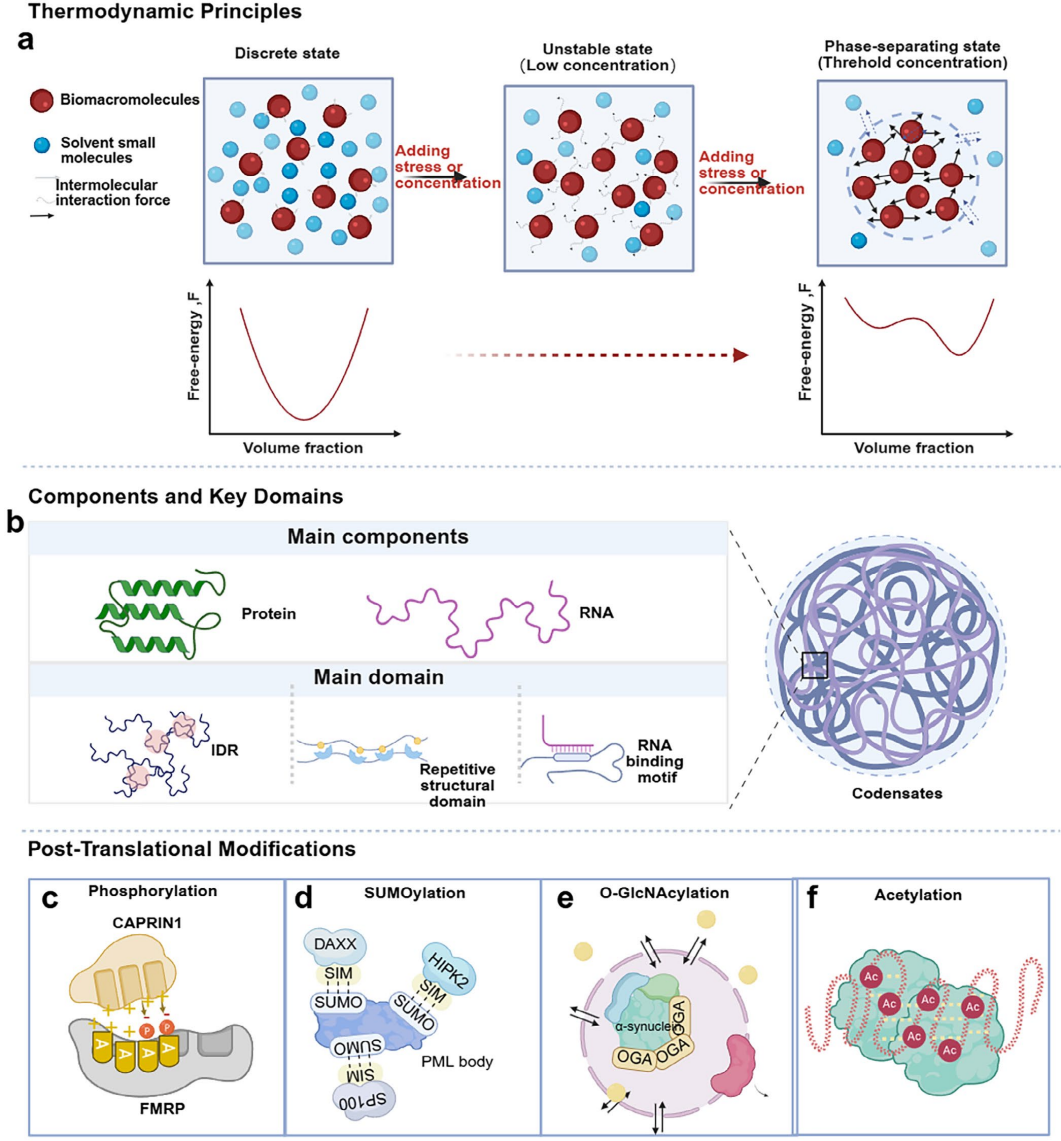
A glance at LLPS. (a) When biomacromolecules are mixed with small‐molecule solvents in the cytoplasm or nucleoplasm, their interactions are typically weak, transient, and nonspecific. Under such low‐affinity molecular interactions, these macromolecules tend to distribute uniformly throughout the solution, exhibiting a discrete state, where the free energy curve is unimodal. When stress is applied to the system or the concentration of biomacromolecules in the solution increases (but remains below the threshold), the intermolecular interactions strengthen, driving the system into an unstable state that is unfavourable for maintaining equilibrium. At this stage, the free energy curve becomes nonmonotonic, displaying a local minimum and forming a biphasic curve. Further increasing the stress or biomacromolecule concentration enhances intermolecular interactions to reach the phase separation threshold, leading to phase separation. This results in the formation of a biomolecule‐rich dense phase and a dilute surrounding solution, driving the system towards a lower free energy state and greater stability. (b) In cells, the primary components of biomolecular condensates are proteins and RNA. Among proteins capable of phase separation, IDRs, repetitive binding domains, RNA‐binding motifs or other structures that mediate multivalent intermolecular or intramolecular interactions are generally considered the decisive structural features driving phase separation. (c) The negatively charged phosphate groups in RNA neutralise the positive charges carried by the arginine‐rich regions of FMRP, thereby reducing electrostatic repulsion with the positively charged CAPRIN1 and promoting their interaction. (d) PML NBs utilise SUMOylated proteins as a core scaffold to recruit client proteins containing SIMs. (e) O‐glycosylation of α‐synuclein enhances the fluidity of condensates by blocking critical protein aggregation events and inducing the formation of novel aggregate structures. (f) In the presence of BRD4‐containing proteins, highly acetylated chromatin forms a novel phase‐separated state, generating droplets with distinct compositions and dynamics.

When environmental factors, such as increased macromolecular concentration or cellular stress, enhance intermolecular attraction, the stability of the homogeneous state may be compromised. Specifically, when solute–solute attraction exceeds solute–solvent interaction, Δ*H*_mix_ becomes negative. In such cases, the enthalpic gain from molecular association can offset the entropic penalty, driving the system away from homogeneity. Under these conditions, the total free energy curve becomes non‐convex. Within a certain concentration range, two‐phase coexistence states, corresponding to local minima of the free energy, are thermodynamically more favourable than the homogeneous state, leading to phase separation. The corresponding chemical potential curve exhibits an inflection with an ‘S’‐shaped profile, losing monotonicity, such that the same chemical potential can correspond to two distinct component concentrations. This allows the system to decompose into two phases with different compositions but a lower overall free energy, thereby realising phase separation [11, 37] (Figure 2a).

However, although classical thermodynamic principles such as free energy minimisation provide a useful framework for understanding LLPS, living cells are inherently nonequilibrium systems in which phase separation behaviours are influenced by multiple complex factors. For instance, macromolecular crowding in the cellular environment profoundly affects LLPS through two distinct mechanisms [38]. One is segregative phase separation, in which crowding‐induced excluded volume effects increase the free energy of macromolecules existing as dispersed monomers, while relatively reducing the entropic penalty associated with aggregate formation. The other is associative phase separation, whereby certain crowding agents, such as polyethylene glycol and dextran, can form favourable interactions with biomacromolecules, lowering the enthalpic term and thereby driving condensate formation to minimise the system's free energy [39, 40]. Notably, in contrast to the static condensates ultimately dictated by thermodynamics, many intracellular phase‐separated assemblies, such as SGs, rapidly form under stress conditions and dissolve promptly upon stimulus removal, indicating that their phase behaviour is maintained as a dynamic steady state rather than a thermodynamic end state [41].

### 2.2 Components of Phase Separation: Scaffold and Client Models

Biomolecular condensates often consist of tens to hundreds of distinct proteins and RNAs [11, 42]. Banani et al. proposed a simplified model to describe the organisational principles of these complex assemblies, distinguishing between multivalent scaffolds and low‐valence clients [18].

Scaffolds act as the primary drivers of phase separation by forming a sufficient number of intermolecular connections per unit volume—either through self‐association or interaction with another protein or RNA—thus promoting LLPS, a process referred to as nucleation of the condensate [43]. Despite constituting a minor fraction of the total content, scaffolds are essential for condensate stability and function and are therefore designated as high‐confidence MLO‐associated proteins [18].

Clients are recruited in a regulated manner through interactions with scaffolds to support condensate growth. This recruitment occurs only at specific stages and is highly dynamic. Clients exhibit significantly higher diffusion rates within condensates compared to scaffolds, reflecting the transient and dynamic nature of scaffold–client interactions [44]. The high valency of scaffolds underlies their ability to recruit other molecules and serves as a key criterion for distinguishing their functional role. However, this distinction is neither static nor absolute; when client valency approaches that of scaffolds, the distinction becomes blurred and clients may begin to compete with scaffolds for interactions [18]. A typical example is provided by RNA‐binding proteins such as TDP‐43. Under physiological conditions, these proteins often act as transient, low‐abundance clients recruited into SGs nucleated by core factors including G3BP1. In disease models, however, pathogenic changes, such as enhanced phosphorylation of TDP‐43 at Ser409/410, increase their local concentration and multivalency in SGs, driving a shift towards scaffold‐like behaviour. This transition is accompanied by remodelling of interaction networks and alterations in condensate physical properties, and can promote pathological self‐assembly and inclusion formation in amyotrophic lateral sclerosis (ALS) [45].

The recruitment of client proteins is governed by multiple, coordinated mechanisms. The prevailing view claims that residue–residue contact propensities, particularly π–π and cation–π interactions, play a dominant role in driving the formation of phase‐separated systems. For example, the prion‐like domain (PLD) of FUS is enriched in polar and aromatic residues, where tyrosine can engage in cation–π interactions with arginine residues within the RNA‐binding domain (RBD), thereby stabilising PLD–RBD associations and forming the basis for scaffold protein assembly [46]. However, recent studies have proposed that the desolvation energy determined by the amino acid composition of the client protein also plays a critical role in client partitioning, which reflects the thermodynamic propensity of a protein to transition from a dilute aqueous phase into a protein‐rich dense phase. In the case of the nuclear pore complex, the partition coefficient of client proteins shows a strong positive correlation with the number of aromatic residues they contain, while this correlation is independent of the amino acid composition of other proteins within the condensate [47]. This observation indicates that the principal driving force for aromatic residues to enter the dense phase arises from their hydrophobicity‐mediated interfacial affinity, rather than from specific residue–residue pairings [47].

Recruitment efficiency also depends on scaffold stoichiometry and client valency, especially when specific interaction sites are present. When scaffold sites are in excess, they facilitate client accumulation through available binding interfaces. In contrast, when scaffold stoichiometry is insufficient, scaffold–scaffold interactions saturate these binding sites and thus limit access to homologous client proteins [18].

Selective proteolytic cleavage or covalent modifications of scaffolds can modulate both stoichiometry and valency, thereby offering a means to control condensate composition and even regulate its formation or dissolution [18]. In addition, the flexibility of scaffold regions further enhances condensate function, as demonstrated in synthetic kinase condensates [48].

### 2.3 The Driving Forces and Key Domains of LLPS

The sticker–spacer model offers critical insights into the structural foundations of phase separation. In this model, ‘stickers’ refer to specific motifs within biomacromolecules that mediate transient and reversible attractive interactions. These interactions form physical crosslinks both intra‐ and intermolecularly, driving condensation behaviours such as LLPS [49]. The establishment of multivalent crosslinks—known as multivalent interactions—constitutes a fundamental driving force in the formation of dynamic, reversible molecular networks that underpin phase‐separated states [50, 51]. Multivalent interactions in biomolecules arise from intrinsically disordered regions (IDRs) and folded domains.

IDRs are protein segments lacking a stable three‐dimensional structure under physiological conditions. These regions are often categorised as low‐complexity domains (LCDs), PLDs or amyloidogenic cores on the basis of their compositional features. LCDs are particularly characterised by amino acid enrichment patterns, mediate electrostatic, π–π and hydrophobic interactions—which are frequently observed in phase‐separating proteins [52, 53, 54].

Owing to their lack of a defined conformation, IDRs behave as dynamic ensembles of rapidly interconverting conformations, similar to the inherently dynamic nature of biomolecular condensates. The conformational plasticity of IDRs is highly sensitive to environmental conditions, including temperature, ionic strength, posttranslational modifications (PTMs) and pH, rendering IDRs central regulators and promising therapeutic targets in diseases linked to abnormal condensation.

The structural instability of IDRs also contributes to condensate maturation—a process marked by a gradual loss of liquid‐like dynamics [55], which is considered driven by the transition of disordered conformations into β‐sheet‐rich, amyloid‐like fibrillar structures [56]. Thus, IDRs exhibit a functional dichotomy, acting as dynamic scaffolds in physiological contexts while serving as pathological aggregation cores under disease conditions. For example, the LCD of the RNA‐binding protein hnRNPA1 facilitates its LLPS with itself and other associated RNA‐binding proteins, promoting the dynamic assembly of SGs. However, disease‐associated mutations within the LCD, such as the pathogenic D262V mutation in hnRNPA1, enhance the propensity of LCDs to adopt stable fibrillar conformations, resulting in persistent SG formation and pathological fibrillisation. Such aberrant phase behaviour contributes to the pathogenesis of neurodegenerative diseases. Importantly, recent studies have highlighted that these LCDs inherently possess a transient fibrillisation tendency even under physiological conditions. These labile fibrillar states are believed to provide mechanical stabilisation within condensates, supporting their functional architecture while preserving dynamic exchange. However, mutations associated with neurodegenerative diseases significantly amplify the intermolecular β‐sheet interactions within LCDs, converting these reversible, metastable fibrillar networks into persistent, pathological aggregation cores [41].

Notably, new research shows that such fibrillisation is not limited to IDRs; the RNA recognition motif (RRM) of FUS can undergo amyloid‐like transformation upon partial unfolding [57]. Dysregulated maturation, including mutant p53 aggregation in cancers and misfolded protein deposition in neurodegenerative diseases, is implicated in disease pathogenesis [54, 58].

IDRs also mediate subcellular targeting. For example, TIA1's PLD drives SG recruitment and FUS's N‐terminal IDR supports RNA‐rich condensate localisation [57, 59].

Structured interaction domains, by contrast, engage in defined, high‐affinity interactions via modular domain recognition. One major mechanism through which structured domains enhance their valency and phase‐separated ability is oligomerisation [52]. A notable example is NPM1, which forms pentamers through oligomerisation of its folded N‐terminal domain, effectively increasing its valency to 10 [35, 60]. Furthermore, some proteins acquire emergent multivalence through higher‐order oligomerisation. Specifically, these assemblies not only expose more interaction sites but also increase their spatial concentration, promoting interactions between adhesive regions [53]. For example, in TDP‐43, the complex heterotypic interactions among N‐terminal domain (NTD)‐RRM1, NTD‐C‐terminal domain (CTD) and RRM2‐CTD structures form an intricate interdomain interaction network and thus its phase separation behaviour [61, 62].

Structured interaction domains and IDRs often act synergistically to facilitate LLPS, as RNA‐binding proteins such as hnRNPA1 provide illustrative examples. While the LCD of hnRNPA1 alone is sufficient to mediate LLPS, the RRMs facilitate phase separation in the presence of RNA by lowering the critical concentration required. It is hypothesised that RNA provides multivalent binding sites that engage in both homotypic and heterotypic interactions with IDRs, forming larger oligomers through two distinct forms of multivalency [57] (Figure 2b).

To dissect LLPS mechanisms, techniques such as all‐atom simulations and coarse‐grained modelling have been applied to probe biomolecular behaviour [51, 63, 64]. A representative example is the use of coarse‐grained modelling to elucidate the scaffold–client interactions within CBX2–RING1B condensates. In this study, AlphaFold‐predicted structures of CBX2 and RING1B were converted into residue‐level coarse‐grained models, where each amino acid is represented by a bead characterised by its charge, hydrophobicity, mass and van der Waals radius. Using direct‐coexistence simulations, both the dilute and condensed phases were simulated within a single system to capture spontaneous phase separation. The results revealed that electrostatic interactions between specific domains of CBX2 play a central role in condensate formation and successfully recapitulated the system's re‐entrant phase behaviour by varying component concentrations [65].

Additionally, LLPS‐specific protein databases—LLPSDB, DrLLPS and PhaSePro—catalogue known phase‐separating proteins and interaction motifs, serving as vital resources for experimental and computational studies [66] (Table 1). These databases systematically catalogue experimentally validated phase‐separating proteins, interaction motifs and functional annotations. For instance, DrLLPS integrates computational tools that allow researchers to predict IDRs and domain features in proteins like FUS, providing a structural foundation for further modelling and mechanistic exploration of LLPS.

**TABLE 1:** Main LLPS‐related database and predictive tool.

Year	Name	Description	Object	Access link
Database				
2019	DrLLPS	Information on LLPS‐related proteins	Protein	https://llps.biocuckoo.cn
2019	HUMAN CELL MAP	Comprehensive information on LLPS‐related proteins	Protein	https://cell‐map.org/
2019	RNAgranuleDB	Literature evidence supporting gene or protein association with the stress granules and P‐bodies	Comprehensive	http://www.rnaphasep.cn
2020	LLPSDB	Information on LLPS‐related proteins and their corresponding experimentally validated phase separation conditions	Protein	http://llpsdb.org
2020	PhaSePro	Comprehensive information of LLPS‐related proteins	Protein	https://phasepro.elte.hu/
2020	PhaSepDB	Comprehensive information of LLPS‐related proteins	Protein	http://db.phasep.pro/
2020	PhaSeDis	Data of LLPS and MLOs associated with various diseases	Comprehensive	http://mlodis.phasep.pro/
2021	RNAPhaSep	Information on LLPS‐related RNAs and their corresponding experimentally validated phase separation conditions	RNA	http://www.rnaphasep.cn
2022	RPS	Provide comprehensive information on LLPS‐related RNA	RNA	http://rps.renlab.org
2023	CD‐CODE	Comprehensive information on biomolecular condensates	Protein	https://cd‐code.org/
Tools for prediction				
2012	D^2^P^2^	Pre‐computed disorder predictions on proteins from completely sequenced genomes	Protein (IDR)	https://d2p2.pro/
2019	PSPer	An in silico screening tool for prion‐like RNA‐binding phase‐separation proteins	Protein	https://www.bio2byte.be/b2btools/psp/
2021	DeePhase	Prediction on protein phase separation	Protein	https://deephase.ch.cam.ac.uk/
2021	DisPhaseDB	Prediction on disease‐related variant protein data that can undergo LLPS	Protein	http://disphasedb.leloir.org.ar
2022	PSPredictor	Supporting query and prediction of potential phase separation proteins	Protein	http://www.pkumdl.cn/PSPredictor
2022	FuzDrop	Prediction on the probability of proteins to undergo LLPS	Protein	https://fuzdrop.bio.unipd.it/

### 2.4 The Regulation of Phase Separation: PTMs

Biomolecular phase separation is regulated through two main mechanisms: modulation of macromolecular solubility and changes in valency or conformation [67]. In the latter, PTMs are central regulators that modulate intermolecular interactions through the covalent addition of functional groups [53].

Phosphorylation is the most extensively studied PTM in LLPS regulation. The addition of phosphate groups alters the charge distribution, which may either promote or inhibit condensate formation. For instance, the phosphorylation of FMRP enhances its interaction with CAPRIN1 (Figure 2c), whereas phosphorylation of FUS leads to condensate dissolution [68, 69]. Besides, N‐terminal phosphorylation of HP1α promotes droplet formation and induces a conformational change from a compact to an extended dimer, highlighting that phosphorylation can further alter protein conformation [70, 71].

SUMOylation represents another regulatory mechanism that promotes intermolecular multivalent interactions. Specifically, SUMOylation involves the covalent attachment of SUMO proteins to lysine residues via isopeptide bonds, thereby facilitating new interactions with proteins harbouring SIMs. In PML nuclear bodies, SUMOylated PML recruits SIM‐containing proteins such as DAXX, HIPK2 and SP100 [72] (Figure 2d).

Other PTMs, such as acetylation and O‐GlcNAcylation, influence condensate material properties. For example, O‐GlcNAcylation increases fluidity and inhibits fibrillisation (Figure 2e), whereas acetylation of chromatin‐associated proteins drives the formation of dynamic phase‐separated droplets with distinct compositional features [73, 74] (Figure 2f).

In pathological contexts, aberrant PTMs of biomacromolecules can modulate the formation, dissolution or properties of condensates, thereby rewiring key signalling pathways and contributing to tumorigenesis, often in coordination with metabolic reprogramming. One notable example involves the tumour‐associated metabolic shift towards elevated lactate levels. AARS1, acting as an intracellular lactate sensor, mediates site‐specific lactylation of p53 within its DNA‐binding domain. This modification significantly impairs p53's DNA‐binding affinity and reduces its efficiency in forming condensates, ultimately attenuating its tumour suppressor function [75]. Another example is observed in melanoma, where purinosome assembly is required to support cellular proliferation and tumour growth. This process depends on ASB11‐mediated polyubiquitination of PAICS, a key purine biosynthetic enzyme. Polyubiquitinated PAICS recruits the ubiquitin‐associated protein UBAP2, which in turn facilitates LLPS and purinosome assembly, thus triggering purinosome assembly [76].

## 3 LLPS in Hallmarks of Cancer

Given the critical role of phase separation mechanisms in regulating normal cellular biochemical activities, aberrant phase separation and its resulting products can lead to pathological states, as prominently exemplified in the acquisition of cancer hallmarks.

### 3.1 Sustaining Proliferative Signalling

The acquisition of autonomy in growth signalling represents a fundamental step in sustaining cancer cell proliferation and driving malignant transformation. This autonomy is achieved primarily through oncogenic mutations that result in constitutive activation of downstream components within growth signalling pathways, enabling proliferation independent of exogenous growth factor stimulation [1, 2].

Chromosomal rearrangements involving receptor tyrosine kinases (RTKs) such as ALK and RET often eliminate their transmembrane domains while preserving their intracellular signalling domains. These intracellular domains can fuse with the N‐terminal fragment of EML4 through phase separation‐mediated mechanisms, resulting in the assembly of oncogenic RTK fusion protein condensates, such as EML4–ALK. These condensates nucleate in a membrane‐independent manner and locally concentrate downstream signalling components, thereby activating RAS/MAPK signalling. Notably, we observed a localised enrichment of both PI3K regulatory (p85) and catalytic (p110β) subunits within RTK fusion protein condensates. However, cellular PI3K activity, as measured by fluorescent reporter, remains concentrated at the plasma membrane rather than within EML4–ALK condensates. This indicates that PI3K/AKT pathway activation continues to rely on lipid‐associated membrane microenvironments for catalytic function [77]. In this condition, condensates may serve as pre‐assembled scaffolding hubs, priming PI3K complexes for more efficient membrane engagement and subsequent downstream activation. This mechanism is conceptually analogous to the pre‐clustering of LAT scaffolds in T‐cell receptor (TCR) signalling, which enhances signal propagation kinetics upon receptor activation [78]. These observations suggest that phase‐separated condensates act as spatial organisers, orchestrating the assembly and readiness of signalling complexes to coordinate with membrane‐resident platforms, thereby fine‐tuning the spatial and temporal dynamics of growth signalling networks.

Another means by which cancer cells sustain proliferation is by circumventing growth‐inhibitory checkpoints. Under stress conditions, the RNA‐binding protein RBFOX2 translocates from the nucleus to the cytoplasm and is sequestered into SGs. Within SGs, RBFOX2 co‐localises with RB1 mRNA, suppressing its translation and promoting unchecked cell cycle progression. In vivo studies have demonstrated that resveratrol induces AMPK activation, which subsequently mediates an increase in the phosphorylation level of Rbfox2, inhibiting its translocation from the nucleus to the cytoplasm and its localisation in SGs, thereby restoring transcription and translation of genes involved in cell cycle regulation. The underlying mechanism may be associated with the regulation of RBFOX2 nucleo‐cytoplasmic trafficking behaviour by its phosphorylation modification. In a mouse melanoma model, resveratrol administration significantly suppressed tumour growth, accompanied by the restoration of RBFOX2 nuclear localisation and increased RB1 protein expression, highlighting the therapeutic potential of modulating protein subcellular localisation to intervene in the dynamics of pathological condensates [79].

Beyond SG‐related interventions, recent studies have also identified other drugs targeting pathological condensates that exhibit promising therapeutic efficacy in suppressing tumour growth in vivo. Nilotinib was found to inhibit tumour growth in osteosarcoma by suppressing the formation of WDR3‐mediated phase‐separated condensates. These studies provide novel strategies at the level of cancer cell proliferation for targeting LLPS‐related oncogenic mechanisms [80].

### 3.2 Enabling Replicative Immortality

A defining feature of cancer cells is their ability to bypass telomere shortening‐induced replicative senescence or apoptosis, thereby achieving replicative immortality. This process is accomplished through two major telomere maintenance mechanisms (TMMs): telomerase reactivation and alternative lengthening of telomeres (ALTs). Recent studies suggest that both mechanisms are tightly coordinated through LLPS, which organises key components into dynamic, membraneless nuclear condensates that support telomere elongation.

#### 3.2.1 LLPS and Telomerase Reactivation

Telomerase reactivation is the predominant TMM in most human cancers [81, 82]. Telomerase, a ribonucleoprotein complex, consists of the catalytic subunit hTER, which prevents telomere erosion by using the telomerase RNA (hTR) template to add six‐nucleotide repeats to the 3′ end of chromosomes via reverse transcription [81, 82, 83].

The recruitment of telomerase to telomeres in cancer cells is facilitated by CBs, which are dynamic nuclear compartments regulated by LLPS. Specifically, the CAB box within the hTR terminal loop interacts with the CB‐resident protein TCAB1, anchoring telomerase to CBs [84, 85]. Knockout of TCAB1 or Coilin significantly disrupts this localisation, resulting in telomere shortening [86]. Additionally, TCAB1 localises to telomeres in a telomerase‐dependent, but Cajal body‐independent manner, facilitating the direct transport of telomerase to telomeres [86]. Using RNA interference to eliminate TCAB1 disrupts the telomerase‐telomere interaction and abolishes telomerase‐mediated telomere synthesis [87, 88].

#### 3.2.2 LLPS in ALT Mechanisms

In tumours with absent or low telomerase activity—such as osteosarcomas, soft tissue sarcomas and gliomas—the ALT pathway serves as the dominant TMM [89]. ALT relies on homologous recombination‐mediated telomere elongation and is supported by the formation of ALT‐associated promyelocytic leukaemia bodies (APBs) [90, 91]. APBs are not passive aggregates, but phase‐separated, dynamic biochemical reaction centres, which are assembled through multivalent interactions between SUMOylated proteins and their interacting SIM proteins [90, 92]. These interactions drive the assembly of dynamic structures that aggregate critical components of the ALT process, including telomeric DNA, replication proteins and DNA helicases such as BLM and ATRX/DAXX. Within APBs, LLPS creates a unique microenvironment that facilitates critical steps of the ALT pathway, such as homologous recombination and mitotic DNA synthesis [90, 93]. The shelterin complex, a multiprotein assembly tightly associated with telomeres, also participates in ALT‐mediated telomere maintenance through LLPS [94]. Core components such as TRF1 and TRF2 bind specifically to telomeric double‐stranded DNA and promote the condensation of telomeric chromatin. This phase‐separated structure serves as a spatial hub that selectively recruits DNA repair factors and telomere‐associated proteins, coordinating telomere extension in ALT cells via dynamic remodelling of chromatin states [94, 95, 96].

Recent findings highlight the critical role of non‐coding telomeric repeat‐containing RNA (TERRA) in promoting and stabilising LLPS at telomeres [97]. TERRA plays a critical role in the formation of R‐loops, which facilitate homology‐directed DNA synthesis, a key step in the ALT pathway. Besides, TERRA's tandem TTAGGG repeats promote the formation of G‐quadruplex (G4) structures, which act as molecular scaffolds that increase the recruitment and organisation of essential components involved in LLPS‐driven processes for telomere maintenance [98]. The lysine‐specific demethylase LSD1 interacts with TERRA via its G4 motif and undergoes co‐phase separation, which amplifies R‐loop‐driven recombination and telomere extension [98, 99]. This illustrates how RNA structure and LLPS dynamics intersect to enable telomere homeostasis in ALT‐positive cancers.

### 3.3 Activating Invasion and Metastasis

The acquisition of invasive and metastatic capabilities marks a critical transition towards advanced cancer stages. These processes involve a cascade of events—including local invasion, intravasation, dissemination as circulating tumour cells (CTCs) and colonisation at distant organs—culminating in secondary tumour formation [100, 101]. LLPS and the dynamic biomolecular condensates it mediates may provide valuable insights into the dynamic and complex mechanisms underlying tumour invasion and metastasis.

#### 3.3.1 Role of LLPS in Tumour Cell Invasion

Modulation of intercellular adhesion properties—such as the regulation of tight junctions (TJs), cadherin phenotype switching or degradation of the extracellular matrix (ECM)—represents critical early steps of local invasion, enabling tumour cells to detach from the primary tumour mass and invade the surrounding stroma, thus acquiring the high motility and invasiveness required for metastasis.

Emerging evidence highlights the role of phase separation in coordinating TJ regulation through the compartmentalisation of key adhesion molecules. TJs function as molecular barriers at the cell surface, restricting diffusion between the apical and basolateral membrane domains and controlling intercellular molecular and ion passage, thus maintaining apicobasal polarity [102, 103]. Early in invasion, TJ proteins are often downregulated, which is associated with the loss of polarity. ZO‐1, a key TJ scaffold protein, forms membrane‐tethered condensates via LLPS, which organises adhesion receptors, cytoskeletal adapters and signalling molecules. This phase behaviour supports continuous TJ assembly and apicobasal polarity. Notably, ZO‐1 is frequently downregulated during tumour invasion and metastasis and given its established role in condensate‐mediated TJ organisation, it is reasonable to infer that reduced ZO‐1 expression may impair its condensate‐forming capacity, weaken intercellular adhesion and thereby facilitate invasion [104, 105]. However, definitive evidence establishing the temporal and mechanistic causality of this process remains to be elucidated. ZO‐1 phase separation‐deficient mutants or live‐cell imaging of condensate dynamics during invasion may be beneficial for further exploration.

Integrin adhesion complexes (IACs), another key structure in TJs, link the ECM to the cytoskeleton and serve as signalling platforms [106]. IAC formation relies on LLPS‐driven dynamic assembly. IACs initially form as nascent adhesions within lamellipodia. Studies have shown that under phosphoinositide signalling, integrin‐associated proteins are recruited from the cytosol to the membrane and interact with β‐integrin cytoplasmic domains, promoting integrin cluster formation and enabling rapid component exchange between condensates and the cytoplasm [107, 108]. These condensates act as hubs to recruit other essential components for IAC assembly. In vitro models by Case et al. further demonstrated that co‐phase separation driven by p130Cas and FAK underlies nascent adhesion formation, with Kindlin coupling Cas‐ and FAK‐dependent LLPS to integrins as a central mediator [109].

In addition, LLPS participates in IAC maturation through PTMs. Paxillin (PXN), a key FA scaffold protein, facilitates FA maturation by recruiting and sequestering FA‐resident proteins at nascent adhesion sites while enhancing downstream signalling via the local enrichment of key signalling components [110]. ULK1/2 have been identified as negative regulators of cellular mechanosensation, and their inhibitory effects on cell migration are dynamically modulated by ECM stiffness. Under high‐stiffness conditions, ULK1/2 attenuate FA‐mediated mechanotransduction, characterised by impaired FA assembly and maturation, suppressed actin cytoskeletal remodelling and reduced cellular tension and stiffness. Mechanistically, ULK1/2 phosphorylate PXN at serine residues S32 and S119, disrupting its homotypic interactions and LLPS behaviour and thus inhibiting PXN‐driven FA formation and the cellular response to mechanical stimulation. Notably, ULK1/2 antagonise the FAK within the PXN signalling axis. Serine phosphorylation of PXN by ULK1/2 interferes with tyrosine phosphorylation events mediated by FAK/Src, thereby suppressing downstream pro‐migratory signalling. The analysis of TCGA BRCA data reveals that reduced ULK1/2 expression correlates significantly with poor prognosis [111].

LLPS also drives epithelial‐to‐mesenchymal transition (EMT), a key step in invasion. DDX21 forms nuclear condensates that activate EMT‐associated genes such as MCM5 in colorectal cancer. Deletion of the key IDR of DDX21 abrogates condensate formation and gene activation [112]. Similarly, DAZAP1 promotes EMT in oral squamous cell carcinoma via LLPS‐mediated regulation of alternative splicing and COX16 expression [113].

#### 3.3.2 Role of LLPS in Tumour Cell Metastasis

Cancer cell migration requires dynamic cytoskeletal remodelling, facilitated by actin–microtubule interactions [114]. Sun et al. reported that cancer cells perceive solid stress through calcium ion‐dependent phase separation of the cytoskeleton‐associated protein CKAP4, which subsequently mediates microtubule branching and lamellipodia formation via the interaction between CKAP4 condensates and microtubules [115]. Furthermore, filamin serves as a physiological crosslinker to condense dispersed F‐actin into spindle‐shaped droplets, thereby mediating changes in cytoskeletal morphology and dynamics [115, 116].

During migration, cells form lamellipodia by pushing the plasma membrane outward, a process requiring localised actin polymerisation and depolymerisation. At the leading edge, phosphorylation‐dependent condensates involving Nephrin–LAT–NCK–N‐WASP activate Arp2/3 and promote F‐actin accumulation, thus driving the formation of lamellipodia and prolonging the cell membrane [117].

In bone metastasis, LLPS regulates tumour dormancy and colonisation. DACT1, which is induced by TGF‐β, acts as a cytoplasmic scaffold that forms condensates to sequester CK2, a Wnt pathway activator. By inhibiting Wnt signalling, DACT1 maintains tumour dormancy and suppresses osteolytic outgrowth. DACT1 deletion in murine models markedly delays metastatic progression, underscoring its regulatory role [118].

### 3.4 Avoiding Immune Destruction

Tumour immune evasion encompasses diverse mechanisms within the tumour microenvironment (TME) that enable malignant cells to avoid immune detection and destruction. These include the recruitment of immunosuppressive inflammatory cells, such as regulatory T‐cells and myeloid‐derived suppressor cells, and the establishment of a suppressive microenvironment.

In physiological immune responses, LLPS promotes the spatial organisation of signalling molecules, facilitating effective immune activation. For instance, in TCR signalling, TCR activation induces LAT phosphorylation, leading to the recruitment of adaptor proteins such as Grb2 and SOS1 into phase‐separated clusters at the immune synapse [78, 119]. Similarly, B cell activation involves triple phase separation among SLP65, CIN85 and lipid vesicles, whereas the transcription factor EBF1 utilises phase separation to facilitate chromatin remodelling during B cell differentiation [120].

**FIGURE 3:**
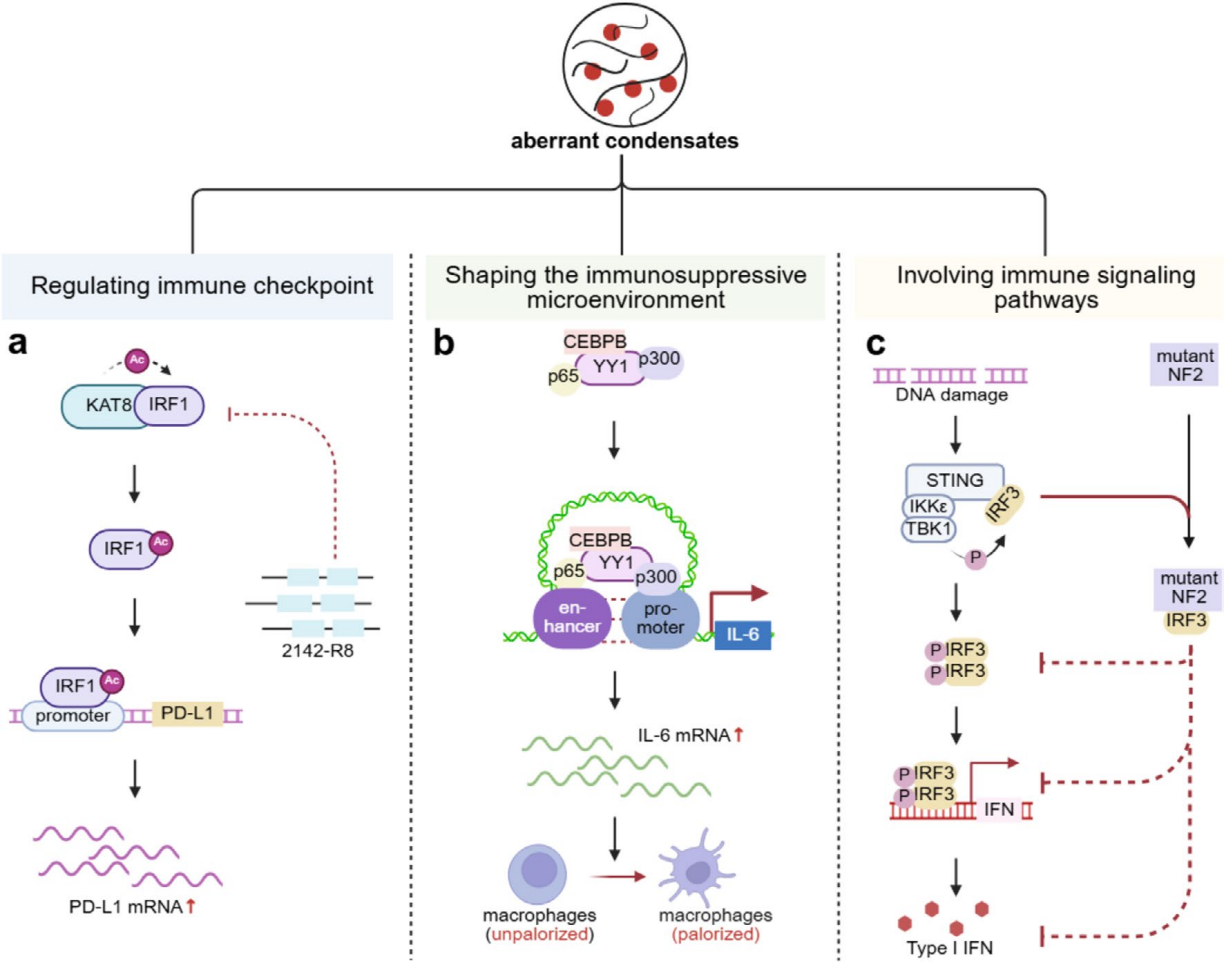
Aberrant condensates in evading immune destruction. (a) KAT8‐IRF1 facilitates the acetylation of IRF1 at K78. This acetylation enhances IRF1 binding to the PD‐L1 promoter and further enriches the transcriptional machinery to promote PD‐L1 mRNA expression. Disruption of the KAT8‐IRF1 phase‐separated condensate with the competitive peptide 2142‐R8 inhibits PD‐L1 expression. (b) The transcription factor YY1 promotes condensates with p65, p300 and CEBPB. These condensates serve as structural regulators in enhancer–promoter loops that upregulate IL‐6 expression, which promotes M2 macrophage polarisation. (c) Upon DNA damage, the STING signalosome assembles and recruits TBK1 and IKKε, leading to their activation. These kinases subsequently phosphorylate and activate IRF3. Phosphorylated IRF3 undergoes dimerisation and functions as a transcription factor to induce type I interferon gene expression. Notably, mutated NF2 condenses with IRF3, which abrogates IRF3 activation phosphorylation and consequently suppresses the cGAS‐STING signalling pathway.

Although LLPS coordinates essential immune functions, increasing evidence indicates that tumours hijack this mechanism to evade immune surveillance (Figure 3).

#### 3.4.1 LLPS in Regulating Immune Checkpoints

Recent studies suggest that LLPS is involved in tumour immune evasion by regulating the spatial organisation and function of immune‐related molecules. A pivotal example is PD‐L1, an immune checkpoint protein that is frequently overexpressed in cancer. PD‐L1 interacts with the PD‐1 receptor on T cells, inhibiting cytotoxic T cell proliferation and promoting immune evasion. Recent research by Wu et al. demonstrated that the overexpression of PD‐L1 in tumour cells is associated with multivalent phase separation between the histone acetyltransferase KAT8 and the transcription factor IRF1. Upon exposure to IFNγ, the biomolecular condensate KAT8‐IRF1 forms, facilitating the acetylation of IRF1 at K78. This acetylation enhances IRF1 binding to the PD‐L1 promoter and further enriches the transcriptional machinery to promote PD‐L1 mRNA expression. Disruption of the KAT8‐IRF1 phase‐separated condensate with the competitive peptide 2142‐R8 inhibits PD‐L1 expression and enhances antitumour immunity [121] (Figure 3a).

Conversely, the RNA‐binding protein YTHDF3 promotes the degradation of HSPA13 mRNA via phase separation and the recruitment of DDX6, indirectly downregulating PD‐L1 levels. Loss of YTHDF3 impairs this pathway, reactivating antitumour immune responses in hepatocellular and renal carcinomas [122].

#### 3.4.2 LLPS in Shaping the Immunosuppressive Microenvironment

Another mechanism by which tumours achieve immune evasion is through the recruitment of immune cells or the secretion of suppressive factors to establish an immunosuppressive TME. Macrophages are key players in this process, and the M2 subtype is regarded as the major phenotype in tumour‐promoting [123]. The transcription factor YY1 promotes M2 macrophage polarisation and tumour progression by forming phase‐separated condensates with p65, p300 and CEBPB. These condensates serve as structural regulators in enhancer–promoter loops that upregulate IL‐6 expression [124] (Figure 3b). In addition, the RTK EphA2 forms condensates in colorectal cancer cells and is positively associated with infiltration by macrophages, neutrophils and dendritic cells, suggesting a potential role in shaping the immune landscape [125].

In addition to molecules directly involved in LLPS, Zhang et al. discovered that mutations or aberrant expression of LLPS regulators such as BRD4, FBN1 and TP53 are correlated with immune cell infiltration and prognosis in digestive system tumours. Given the strong prognostic stratification capabilities of these LLPS regulators, they may serve as novel biomarkers for predicting the prognosis and immunotherapy response of digestive system tumours [126].

#### 3.4.3 LLPS in Immune Signalling Pathways

LLPS also plays a critical role in immune signalling pathways, such as the cGAS‐STING pathway, which mediates innate antitumour immunity. cGAS, a DNA sensor, undergoes conformational changes upon DNA binding, triggering phase separation and activation. This results in cGAMP production and STING activation, leading to the recruitment of TBK1 and the subsequent production of type I interferons. However, mutations in the NF2 gene, which encodes neurofibromin 2, disrupt this pathway. In NF2‐mutant tumours, such as vestibular schwannomas, the NF2 FERM domain forms phase‐separated condensates with IRF3, inhibiting TBK1 activation and dampening the antitumour immune response [127] (Figure 3c).

### 3.5 Reprogramming Energy Metabolism

Compared with normal cells, cancer cells exhibit distinct metabolic patterns. While normal cells predominantly rely on oxidative phosphorylation for energy production, tumour cells shift towards aerobic glycolysis—a phenomenon first described by Otto Warburg in the 1920s, known as the ‘Warburg effect’ [128, 129]. This metabolic reprogramming enables rapid ATP generation, sustains survival under hypoxia and supplies essential biosynthetic precursors, including carbon and nitrogen sources for nucleotide and ribosome synthesis [130, 131].

Mitochondrial metabolism is tightly regulated by phase separation events [132]. An increase in the number of PML nuclear bodies enhances the transcriptional activity of PGC‐1α, thereby upregulating mitochondrial respiratory genes and boosting oxidative phosphorylation [133]. In mesenchymal stem cells harbouring SHP2E76K mutations, aberrant phase separation promotes hyperactivation of mitochondrial complexes I and III, driving sarcoma‐like transformation [134]. Additionally, the p53‐induced protein Mieap regulates mitochondrial quality by forming MLOs that compartmentalise cardiolipin metabolism. Loss of Mieap disrupts mitochondrial architecture and contributes to colorectal cancer development [135].

Phase separation facilitates glycolytic flux by assembling multienzyme complexes. The long noncoding RNA NEAT1, which is frequently upregulated in cancers, scaffolds the PGK1/PGAM1/ENO1 enzymes into metabolons that enhance substrate channelling. This organisation increases the efficiency of glycolysis, enabling rapid metabolic adaptation to meet proliferative demands [136].

In hepatocellular carcinoma, downregulation of G6PC leads to intracellular glycogen accumulation. Phase‐separated glycogen droplets scaffold the Laforin‐Mst1/2 complex, sequestering the Hippo pathway kinases Mst1/2. This sequestration activates YAP, promoting tumourigenesis and underscores a direct link between metabolic reprogramming and oncogene activation [137].

Furthermore, tumour cells adapt to changes in tumour microenvironment through metabolic reprogramming. Under hypoxic conditions or metabolic stress, cancer cells assemble G bodies through LLPS, incorporating glycolytic enzymes and RNA scaffolds. G body formation accelerates glycolytic flux, ensuring energy production under stress. Cells deficient in G body assembly exhibit impaired division and survival. In contrast, cells that form G bodies show increased glucose consumption and reduced levels of glycolytic intermediates, suggesting that the formation of G bodies is a key mechanism in modulating the glycolytic rate and promoting the adaptive changes in energy demand of tumour cells [138]. Another notable example is NRF2, a master regulator of oxidative stress, which is activated through phase separation mechanisms under cellular stress. p62‐mediated sequestration of KEAP1 releases NRF2, enabling antioxidant gene transcription and autophagy induction to degrade damaged organelles, collectively sustaining redox homeostasis and promoting tumour cell survival and proliferation [139, 140]. Research by Li et al. revealed that under glucose deprivation, NRF2 upregulates sestrin2, disrupting LLPS‐dependent IGF2BP3‐HK2 mRNA stabilisation, thereby reducing glycolytic flux and promoting survival during nutrient stress and thus protecting cells from apoptosis induced by glucose starvation [141].

The concept of membraneless metabolic compartments revealed the close relationship between metabolic reprogramming and metastasis in cancer cells. Membrane‐less metabolic compartments refer to specialised phase‐separated microdomains formed at the invasive front, enriching enzymes involved in purine metabolism, including IMPDH, GMPS, guanylate kinase 1 and salvage pathway components such as APRT and ADK. These condensates generate localised ATP/GTP pools to sustain cytoskeletal dynamics and directional migration, thus facilitating metastasis [142].

### 3.6 Non‐Mutational Epigenetic Reprogramming

Tumour cells acquire distinct growth advantages not only through genetic mutation but also via nonmutational epigenetic reprogramming. This form of reprogramming remodels cellular phenotypes and fate decisions without altering the underlying DNA sequence, encompassing processes such as DNA methylation, histone modifications, chromatin remodelling and regulation by noncoding RNAs [143, 144].

#### 3.6.1 Histone Modifications

Histone modifications directly regulate local chromatin structure by altering electrostatic interactions between histones and DNA or indirectly by recruiting effector proteins, chromatin remodelling complexes and other chromatin‐associated proteins to influence chromatin structure [145]. These modifications ultimately regulate DNA processes such as transcription, replication and recombination [146]. Aberrant histone modifications can disrupt cellular homeostasis and promote tumourigenesis, among which the most extensively studied histone modifications in the context of cancer are lysine acetylation and lysine methylation [143, 147].

The regulation of histones at different methylation sites results in distinct functional outcomes. For example, monomethylation of H3K4 (H3K4me1) is localised at gene enhancers and strongly activates target gene expression. The histone lysine methyltransferase KMT2D (also known as MLL4) is a key enzyme in this process, catalysing H3K4 monomethylation at enhancers to promote the expression of critical transcription factors such as LIFR and KLF4, which are involved in oncogenic signalling pathways, including the PI3K/Akt pathway and EMT‐related pathways [148, 149]. Li et al. demonstrated that KMT2D‐mediated LLPS, driven by its LCD, assembles membraneless condensates that stabilise WDR5‐KMT2D complexes and promote H3K4me1 deposition [150]. UTX (also known as KDM6A), an H3K27 demethylase, co‐condensates with KMT2D to enhance methyltransferase activity. This conclusion has been validated in both in vitro reconstitution experiments and cellular engineering systems. Furthermore, UTX regulates various histone modifications and long‐range chromatin interactions across distinct genomic regions in a condensate‐dependent manner to maintain proper chromatin states. Mutations disrupting the IDR of UTX impair its tumour‐suppressive functions by destabilising condensate formation [151].

Conversely, histone acetylation generally promotes transcriptional activation by neutralising lysine charges and loosening DNA–histone interactions [143]. In triple‐negative breast cancer, phosphorylated histone deacetylase 6 (HDAC6) forms nuclear LLPS condensates with importin‐β, reshaping the chromatin architecture. This leads to the closure of immune‐related loci and the opening of lipid metabolism genes, ultimately downregulating tumour suppressor pathways (such as ATF3 and NDRG1) and suppressing antigen presentation and TNF‐α signalling [152].

#### 3.6.2 Chromatin Remodelling

Chromatin remodelling is crucial for regulating DNA accessibility and ensuring proper gene expression. Phase separation plays a critical role by compartmentalising chromatin‐associated factors and reorganising the 3D genome architecture.

NUP98‐HOXA9, a common transcription factor fusion in leukaemia, is established by phase separation in a concentration‐ and valence‐dependent manner. NUP98‐HOXA9 induces the formation of abnormal three‐dimensional chromatin structures, leading to the upregulation of oncogenes (such as PBX3 and HOX) through promoter overlap with NUP98‐HOXA9 binding loops [153]. Besides, core histones with hyperacetylated lysine residues interact with bromodomain‐containing proteins such as BRD4, facilitating the formation of phase‐separated chromatin domains that enhance transcriptional activity [73, 154]. Similarly, the BRD4 fusion oncoprotein BRD4‐NUT induces the formation of aberrant chromatin subcompartments, referred to as ‘M’ bodies, through phase separation. These ‘M’ bodies concentrate acetylated active chromatin and exhibit enhanced chromatin interactions, driving the transcriptional upregulation of key oncogenes such as Myc [155, 156, 157].

## 4 Application of LLPS in Cancer Diagnosis and Therapy

**FIGURE 4:**
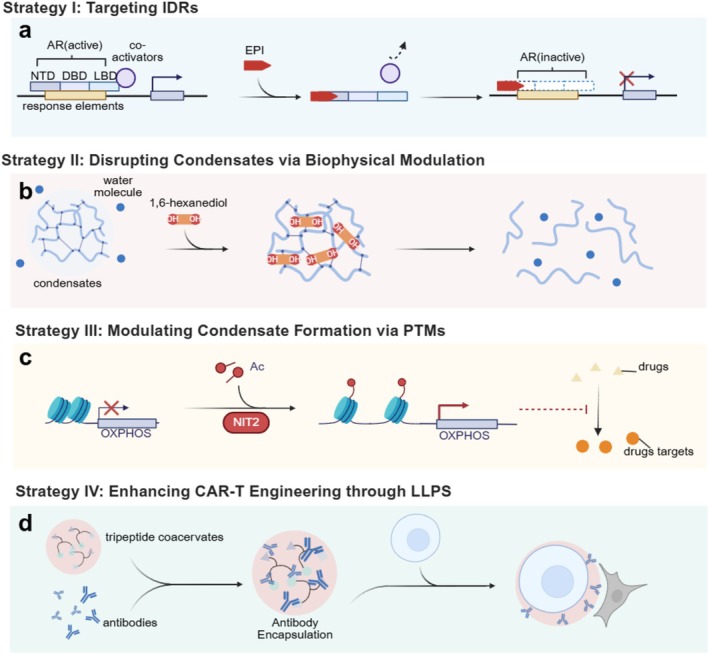
Four LLPS‐targeted therapeutic strategies. (a) The AR is structurally composed of three functional domains: the N‐terminal domain (NTD), the DNA‐binding domain (DBD) and the ligand‐binding domain (LBD). Upon binding to coactivators, AR interacts with androgen response elements on DNA to facilitate the transcription of oncogenic target genes. The EPI series of small‐molecule inhibitors specifically targets the NTD of AR, thereby disrupting AR‐coactivator interactions and subsequently suppressing AR‐driven transcriptional activity. (b) 1,6‐hexanediol dissolves condensates by disrupting weak hydrophobic interactions. (c) The enzyme NIT2 promotes H3K14 acetylation and the upregulation of OXPHOS genes, which are related to drug resistance. Inhibiting NIT2 may restore drug sensitivity by disrupting H3K14 acetylation‐dependent gene expression. (d) In NK cells, synthetic peptides such as Fmoc‐Lys‐Gly‐DOPA‐OH can undergo LLPS to form surface condensate layers that support antibody decoration (such as trastuzumab), enhancing antibody‐dependent cellular cytotoxicity.

Aberrant biomolecular condensates play pivotal roles in cancer progression, driving transcriptional dysregulation, drug resistance, immune escape and metabolic adaptation. These insights have led to the development of new therapeutic strategies that target phase separation directly or leverage its biophysical principles for drug design and delivery. This section highlights recent advances in targeting LLPS‐associated mechanisms (Figure 4), developing biomarker‐driven clinical tools and constructing LLPS‐inspired drug delivery systems.

### 4.1 Therapeutic Targeting of LLPS Mechanisms

#### 4.1.1 Targeting IDRs

Traditionally, cancer drug development has focused on structured protein domains, while IDRs have been deemed ‘undruggable’ owing to their lack of defined tertiary structure [158, 159]. However, recent discoveries have identified IDRs as viable therapeutic targets, particularly in the context of LLPS.

For instance, EPI series small molecule inhibitors covalently bind to the intrinsically disordered NTD of the AR, blocking its interaction with androgen response elements and suppressing AR‐driven transcription in castration‐resistant prostate cancer [31, 160, 161] (Figure 4a). Similarly, inhibitors such as IIA4B20 and Mycmycin‐1/2 target the disordered region of c‐MYC, preventing MYC/Max heterodimerisation and the formation of transcriptionally active condensates [162, 163].

In addition to inhibiting oncogenes, small molecules including ReACp53 [164], tripyridinamide (ADH‐6) [165] and eprenetapopter (APR‐246) [166, 167] modulate the IDR of mutant p53 to disrupt amyloid‐like condensates and restore tumour suppressor activity. Notably, APR‐246 has advanced through phase I to III clinical trials, marking a significant milestone in targeting condensate‐prone proteins [166].

#### 4.1.2 Disrupting Condensates via Biophysical Modulation

In addition to targeting specific protein sequences, some therapies seek to interfere with the physicochemical forces that underlie phase separation. These include hydrophobic interactions, π–π stacking and electrostatic forces, which collectively govern the material properties and dynamics of biomolecular condensates.

Small molecules such as 1,6‐hexanediol, a hydrophobic alcohol, have been widely used to experimentally dissolve LLPS‐driven condensates by disrupting weak hydrophobic interactions. Ming et al. demonstrated that treatment with 1,6‐hexanediol effectively dissolves aberrant condensates in pancreatic cancer models and suppresses tumour cell proliferation [168] (Figure 4b). However, its nonspecificity and cytotoxicity have limited its clinical potential.

To address this, high‐throughput screening methods such as DropScan have been developed to identify compounds that modulate LLPS more selectively. Using this approach, the CDK4/6 inhibitor LY2835219 was found to disrupt EWS‐FLI1‐associated condensates in Ewing sarcoma, rescuing dysregulated gene expression. The underlying mechanism may involve primarily the induction of lysosomal acidification, thereby promoting the colocalisation of condensates with lysosomes [169].

In recent years, an increasing number of studies have indicated that certain naturally derived bioactive peptides possess the potential to target intracellular biocondensates and modulate their phase behaviour. A notable example is the antimicrobial peptide LL‐III, which targets LAF‐1 to induce condensate disassembly and promote the formation of amorphous, non‐fibrillar aggregates. Experimental observations have demonstrated that LL‐III preferentially partitions into LAF‐1 droplets, subsequently disrupting their structure through strong hydrophobic interactions with hydrophobic residue clusters in the LAF‐1 primary sequence, as well as electrostatic interactions with charge patches. This study provides important insights into the use of peptides as tools to regulate LLPS‐associated cellular processes and related diseases [170].

#### 4.1.3 Modulating Condensate Formation via PTMs

PTMs represent a key regulatory layer for controlling the assembly, composition, and dissolution of biomolecular condensates. Targeting PTM‐related enzymes has shown promise in overcoming therapeutic resistance. For instance, the enzyme NIT2 enhances chemoresistance by promoting H3K14 acetylation and upregulating OXPHOS genes. Inhibiting NIT2 may restore drug sensitivity by disrupting H3K14 acetylation‐dependent gene expression [171] (Figure 4c).

Another example involves the SUMO‐specific protease SENP1, which modulates the deSUMOylation of RNF168 and thus inhibits the formation of RNF168 nuclear condensates, thereby facilitating DNA repair and enhancing resistance to genotoxic agents. Pharmacological inhibition of SENP1 has emerged as a potential strategy for sensitising colorectal cancer cells to DNA‐damaging therapies [172].

#### 4.1.4 Enhancing Drug Partitioning and CAR‐T Engineering Through LLPS

The selective partitioning of small molecules into specific biomolecular condensates has emerged as a powerful strategy to enhance drug targeting and efficacy. Small molecules may preferentially localise to condensates on the basis of their hydrophobicity, aromaticity or charge properties, thereby concentrating at the site of their intended action.

For example, tamoxifen accumulates in MED1 condensates, disrupting oestrogen receptor alpha (ERα) condensates and inhibiting the transcription of oestrogen‐responsive genes such as MYC [173]. Cisplatin, enriched with aromatic and polarised functional groups, similarly localises to MED1 condensates via π‐π and π‐cation interactions, enhancing its therapeutic targeting ability [174].

In addition to its ability to be used in intracellular applications, LLPS has also been engineered to improve immune cell therapies. The incorporation of EB6I—a CD3ε cytoplasmic motif—into Chimeric antigen receptor (CAR) constructs induces phase‐separated condensates in the intracellular domain of CAR‐T cells. This clustering enhances antigen receptor signalling and reduces CAR endocytosis, improving tumour cell killing even in low‐antigen environments [175]. In NK cells, synthetic peptides such as Fmoc‐Lys‐Gly‐DOPA‐OH can undergo LLPS to form surface condensate layers that support antibody decoration (such as trastuzumab), enhancing antibody‐dependent cellular cytotoxicity [176] (Figure 4d). These applications exemplify how LLPS‐based engineering can optimise immune cell targeting and persistence in the TME.

### 4.2 LLPS‐Associated Biomarkers in Prognosis and Immunotherapy

The role of LLPS in shaping the tumour immune microenvironment and promoting immune evasion has highlighted its potential as a biomarker for cancer prognosis and immunotherapy stratification. By profiling the expression of LLPS‐associated genes, researchers have identified condensate‐regulating factors that are correlated with immune infiltration, therapeutic response and clinical outcomes (Table 2).

**TABLE 2:** LLPS‐related prognostic biomarkers.

Cancer type	Study type	LLPS‐related genes	Key findings
Pancreatic cancer	Prognostic estimation and risk stratification	PYGB, ACTR3, etc.	Identify 6 LLPS‐related genes. Develop a prognostic risk model (prognosis, immunotherapy).
Prostate cancer	Prognostic estimation, risk stratification and pharmacogenomics	FUS, CBX2, etc.	Identify 6 LLPS‐related genes and 2 subtypes. Develop a prognostic risk model (prognosis, anti‐cancer drugs sensitivity).
Clear cell renal cell carcinoma	Prognostic estimation and pharmacogenomics	HOXA13, TEAD4, etc.	Identify 5 LLPS‐related genes. Develop a prognostic risk model (clinical features, progression, prognosis, immunotherapy, drug sensitivity).
Lower‐grade glioma	Prognostic estimation and risk stratification	225 LLPS‐related genes	Identify 225 LLPS‐related genes. Define 4 subtypes and analyse their differences. Develop a prognostic risk model (prognosis, anti‐cancer drugs sensitivity, immunotherapy).
Skin cutaneous melanoma	Prognostic estimation and risk stratification	MLKL, PARVA, etc.	Identify 6 LLPS‐related genes. Define 2 subtypes and analyse their difference. Develop a prognostic risk model.
Epithelial ovarian cancer	Prognostic estimation and risk stratification	HMBOX1, EIF6, etc.	Identify 11 LLPS‐related genes and reveal their function. Identify 2 subtypes. Develop a prognostic risk model. Use risk score to predict the survival of melanoma patient.
Endometrial cancer	Prognostic estimation and risk stratification	EIF2S2, SNRPC, etc.	Identify 4 LLPS‐related genes. Identify 2 subtypes and analyse their difference; Develop a prognostic risk model.
Breast cancer	Prognostic estimation, risk stratification and mechanistic study	POLR3GL, PLAT, etc.	Identify 9 LLPS‐related genes and 2 subtypes; Identify the biomolecular function of the key gene PGAM1. Develop a prognostic risk model.
Hepatocellular carcinoma	Prognostic estimation, risk stratification, mechanistic study and pharmacogenomics	MAPT, WDR62, etc.	Identify 5 LLPS‐related genes and 2 subtypes. Develop a prognostic risk model (prognosis, immune microenvironment, immunotherapy, drug sensitivity). Identify the biomolecular function of the key gene MAPT.
Lung squamous cell carcinoma	Prognostic estimation	HOXA13, SSX1, etc.	identify 7 LLPS‐related genes and 2 subtypes. Develop a prognostic risk model (prognosis, immune microenvironment, immunotherapy, drug sensitivity).
Colon cancer	Prognostic estimation, risk stratification and mechanistic study	SYN2, POU4F1, etc.	Identify 11 LLPS‐related genes. Identify 2 subtypes and analyse their difference. Develop a prognostic risk model (prognosis, immunotherapy). Validate the biological functions of the key gene POU4F1.
Oesophageal adenocarcinoma	Prognostic estimation, risk stratification and pharmacogenomics	HSPA4 and HNRNPA2B1	Identify 2 LLPS‐related genes and 2 subtypes. Develop a prognostic risk model (prognosis, immunotherapy, drug sensitivity). Speculated on small‐molecule drugs possibly related to LLPS.

Nevertheless, most current studies are based on bioinformatic analyses of publicly available datasets, often with small clinical cohorts. Therefore, validation through in vitro and in vivo models, as well as clinical trials, is essential to confirm the diagnostic and prognostic value of LLPS‐related biomarkers.

### 4.3 LLPS‐Inspired Drug Delivery Systems

Biomolecular condensates formed via LLPS exhibit unique properties such as compartmentalisation without membranes, dynamic responsiveness and tunable permeability—features that have inspired the design of novel drug delivery systems. By mimicking or utilising LLPS principles, researchers aim to improve drug loading, targeting precision and controlled release in cancer therapy.

#### 4.3.1 Synthetic Polypeptoid‐Based LLPS‐Inspired Carriers

Diblock copolypeptoids are synthetic biomaterials designed on the principle of phase separation, capable of undergoing microphase separation in aqueous solutions to yield well‐defined nanostructures such as micelles, vesicles or lamellae. Owing to the absence of hydrogen bond donors and chiral centres, these materials possess excellent solubility, biocompatibility and structural stability. This microphase separation essentially represents an amphiphilic self‐assembly process, resulting in the formation of stable and ordered nanostructures. Although they lack the dynamic liquid droplet behaviour characteristic of conventional LLPS, they recapitulate key advantageous features, including membraneless compartmentalisation and responsiveness to environmental stimuli. These phase separation‐associated properties enable precise spatiotemporal control over drug encapsulation and release [177].

A representative example is provided by redox‐responsive core‐crosslinked micelles, which can encapsulate doxorubicin (DOX) and release it in response to the reducing agent dithiothreitol. This strategy achieves time‐ and concentration‐dependent inhibition of HepG2 tumour cells and demonstrates superior drug utilisation efficiency, as evidenced by greater tumour cell inhibition compared with non‐core‐crosslinked micelles at the same DOX concentration [178]. Similarly, oxidation‐responsive vesicle systems utilising reactive oxygen species (ROS)‐sensitive photoactivatable TPP or H_2_O_2_ exposure can also rupture vesicles to deliver encapsulated agents with spatial and temporal precision [179]. Functionalisation of polypeptide surfaces with targeting ligands, including folic acid or danthronyl, further enables receptor‐specific delivery [177].

Moreover, Lin and Chen and their colleagues have reported crystalline nanoflower‐like particles constructed from fluorinated sequence‐defined peptoids, which exhibit strong nucleic acid‐binding affinity and efficient cytosolic delivery in H1299 and A549 human alveolar basal epithelial adenocarcinoma cells, enhancing the bioavailability and biocompatibility of therapeutic payloads [180].

#### 4.3.2 Drug Delivery System Based on ATPS

Unlike amphiphilic molecular self‐assembly, which is primarily driven by hydrophobic interactions and the minimisation of interfacial energy, ATPS represents a classical form of LLPS arising from the demixing of immiscible polymer–polymer or polymer–salt aqueous solutions. This process produces aqueous compartments that closely mimic cellular microenvironments and exhibit the dynamic liquid droplet characteristics of LLPS [181, 182]. For example, in ATPS vesicles, the contact angle between internal droplets and the membrane can vary with osmotic pressure and polymer concentration, triggering wetting transitions that lead to membrane deformation and budding, which are hallmarks of wetting behaviour [183]. Fusion between the two aqueous phases within ATPS can also occur. In a PEG/dextran ATPS, increasing the temperature can transform the internal ATPS into a single phase, resulting in the redistribution of proteins such as concanavalin A from localised enrichment in the dextran‐rich phase to a uniform distribution within the vesicle interior [184].

Compared with organic solvent‐based emulsions, ATPS‐based carriers avoid protein denaturation, making them ideal for sensitive drug cargoes [33]. Drug encapsulation in ATPS systems can be achieved by directly adding cargo into the ATPS, dissolving the payload in the aqueous solution of the phase‐forming compounds or co‐incubating the carrier with the cargo [184]. ATPS enables selective drug loading on the basis of partitioning affinity. During delivery, dynamic phase exchange permits spatially controlled drug distribution. Environmental triggers—such as acidic pH or elevated ROS levels in tumours—can induce release without the need for exogenous agents [185, 186]. Furthermore, ultrasound‐triggered ATPS capsules have demonstrated localised, dose‐controlled release, whereas interface stabilisation techniques (such as Pickering emulsions and lipid nanocapsules) increase in vivo stability [187].

The bioavailability and tumour‐targeting efficiency of ATPS‐based drug delivery systems have been experimentally validated. For example, Okur et al. encapsulated DOX into CREKA‐functionalised PEG/dextran ATPS‐derived microparticles, achieving sustained release over 144 h [188]. Compared to free DOX, this system exhibited markedly enhanced endocytic uptake in HeLa cells, attributed to the specific binding of the CREKA peptide to collagen IV within the fibrin structures secreted by HeLa cells.

These systems provide a versatile, LLPS‐mimicking platform for next‐generation targeted therapies.

## 5 Conclusion and Perspective

Over the past decade, LLPS has emerged as a unifying principle of biomolecular condensates, bridging polymer physics and cell biology [189, 190]. This framework has expanded our understanding of cellular compartmentalisation beyond membrane‐bound organelles and revealed new layers of regulation in cancer biology.

In this review, we have summarised the molecular and thermodynamic principles of LLPS and illustrated its involvement in nearly all hallmarks of cancer, including proliferative signalling, metabolic reprogramming, immune evasion and metastasis. While substantial progress has been made, many unresolved issues persist. For example, most research has concentrated on single‐phase biomolecular condensates, but this singular approach does not fully capture the complexity of MLOs. The increasing recognition of multiphase condensates provides a more comprehensive model, offering a complete biophysical framework for understanding the spatial organisation, functional partitioning and dynamic behaviour of biomolecules within cells. Experimental evidence, such as oligomerisation‐driven immiscibility, has provided insights into how distinct molecular interactions guide the formation of layered or nested condensates [49]. However, technical limitations in live‐cell detection and functional dissection of these structures still pose major challenges.

In addition, the exploration of the regulatory mechanisms governing biomolecular condensates remains at an early stage. Although factors such as stoichiometry, surface tension and interaction valency are known to influence condensate behaviour, their precise roles in defining function remain incompletely understood. Future studies will need to decipher whether these biophysical parameters can be harnessed to design programmable condensates with therapeutic or synthetic functions.

LLPS also represents a promising avenue in cancer immunotherapy. Novel approaches, such as enhancing CD38 expression via RARα condensates or targeting FOXM1 condensates to increase tumour immunogenicity, demonstrate how condensate modulation can reprogram immune responses [191, 192]. These strategies exemplify the translational potential of LLPS research and highlight its emerging relevance in overcoming immune resistance.

Despite these advances, significant knowledge gaps remain. LLPS mechanisms have yet to be fully integrated across cancer development, from mutational events to epigenetic reprogramming and phenotypic plasticity. Moreover, the heterogeneity of condensates across tumour types, microenvironments and therapeutic contexts demands more systematic and multidimensional profiling. Besides, LLPS behaviour in cancer models, the majority of current evidence remains derived from in vitro systems or limited animal models. With the exception of a few agents (such as EPI‐506) that have entered clinical trials, the translational feasibility of most candidate molecules in vivo requires further validation. Several major challenges hinder clinical translation, including insufficient delivery efficiency and tissue penetration that prevent drugs from reaching target regions in the complex tumour microenvironment. Incorporating nanodelivery systems and controlled‐release platforms may help address this problem. Ensuring targeting specificity and safety to avoid disrupting physiological condensates like SGs or PML bodies is another major concern. For instance, although 1,6‐hexanediol can effectively disrupt certain condensates in vitro, its clinical application is limited by poor stability and low selectivity in vivo.

In conclusion, LLPS provides a conceptual and mechanistic framework for investigating the dynamic molecular organisation of cancer cells. By integrating insights from physics, molecular biology and systems immunology, LLPS research is poised to reshape our understanding of tumour progression and catalyse the development of innovative cancer diagnostics and therapeutics.

## Author Contributions

**Chen‐chen Xie:** conceptualisation, writing – original draft, writing – review and editing. **Ting Wang:** resources, writing – original draft, writing – review and editing. **Xin‐ran Liu:** resources, supervision. **Yan Wang:** supervision, writing – review and editing. **Qin Dang:** supervision, writing – review and editing. **Tian Ding:** resources, supervision. **Jia‐qi Xu:** supervision. **Xian‐jun Yu:** resource, writing – review and editing. **Yi Qin:** project administration, resources, supervision. **Xiao‐wu Xu:** supervision, resources. **Hai Lin:** supervision, resources.

## Ethics Statement

The authors have nothing to report.

## Conflicts of Interest

The authors declare no conflicts of interest.

## Data Availability

Data sharing is not applicable to this article as no datasets were generated or analysed during the current study.
